# Proinflammatory Stimulation of Toll-Like Receptor 9 with High Dose CpG ODN 1826 Impairs Endothelial Regeneration and Promotes Atherosclerosis in Mice

**DOI:** 10.1371/journal.pone.0146326

**Published:** 2016-01-11

**Authors:** Alexander O. Krogmann, Enzo Lüsebrink, Martin Steinmetz, Tobias Asdonk, Catharina Lahrmann, Dieter Lütjohann, Georg Nickenig, Sebastian Zimmer

**Affiliations:** 1 Medizinische Klinik und Poliklinik II, Universitätsklinikum Bonn, 53105, Bonn, Germany; 2 Institut für klinische Chemie und klinische Pharmakologie, Universität Bonn, 53125, Bonn, Germany; Child & Family Research Institute, CANADA

## Abstract

**Background:**

Toll-like receptors (TLR) of the innate immune system have been closely linked with the development of atherosclerotic lesions. TLR9 is activated by unmethylated CpG motifs within ssDNA, but also by CpG motifs in nucleic acids released during vascular apoptosis and necrosis. The role of TLR9 in vascular disease remains controversial and we sought to investigate the effects of a proinflammatory TLR9 stimulation in mice.

**Methods and Findings:**

TLR9-stimulation with high dose CpG ODN at concentrations between 6.25nM to 30nM induced a significant proinflammatory cytokine response in mice. This was associated with impaired reendothelialization upon acute denudation of the carotid and increased numbers of circulating endothelial microparticles, as a marker for amplified endothelial damage. Chronic TLR9 agonism in apolipoprotein E-deficient (ApoE^-/-^) mice fed a cholesterol-rich diet increased aortic production of reactive oxygen species, the number of circulating endothelial microparticles, circulating sca-1/flk-1 positive cells, and most importantly augmented atherosclerotic plaque formation when compared to vehicle treated animals. Importantly, high concentrations of CpG ODN are required for these proatherogenic effects.

**Conclusions:**

Systemic stimulation of TLR9 with high dose CpG ODN impaired reendothelialization upon acute vascular injury and increased atherosclerotic plaque development in ApoE^-/-^ mice. Further studies are necessary to fully decipher the contradictory finding of TLR9 agonism in vascular biology.

## Introduction

Atherosclerosis is a chronic inflammatory disease, characterized by apoptosis and necrosis of vascular cells such as endothelial cells, smooth muscle cells and macrophages. Cellular damage within the vessel wall leads to the release of various compounds including proteins, small molecules and nucleic acids. These danger signals are detected by specialized pattern recognition receptors (PRR) and trigger innate immune mechanisms to promote the clearance of detritus and to engage potential cytotoxic substances [1]. As part of the innate immune system, PRR detect structures of bacterial and viral pathogens, termed pathogen-associated molecular patterns (PAMP), and initiate an intracellular signaling cascade leading to the release of proinflammatory cytokines. One such PRR, Toll-like-receptor (TLR) 9 is located in the endosomes of resident vascular cells, such as endothelial and smooth muscle cells, cardiomyocytes, cardiac fibroblasts, and of professional immune cells, including macrophages, B-cells and plasmacytoid dendritic cells [2–7]. Oligodeoxynucleotides with unmethylated deoxycytidyl-deoxyguanosine dinucleotides (CpG ODN) have been identified as the specific ligand of TLR9 and, although 20x more common in bacterial DNA, are a widespread motif in human DNA [8,9].

So far, pro- and anti-atherosclerotic effects of TLR9 have been described. Koulis et al. have recently reported, that deletion of TLR9 exacerbated atherosclerosis in ApoE^-/-^ mice, and stimulation of TLR9 in ApoE^-/-^ mice led to reduced development of atherosclerotic plaque, implicating a protective function of TLR9 [10]. These unexpected findings oppose our current concept of innate immune activation in atherogenesis, because TLR9 signaling is mediated by the highly proinflammatory adaptor protein myeloid differentiation primary response protein (MyD88). MyD88 is required for the signal transduction of all TLRs, except TLR3 and endosomal TLR4, to initiate a proinflammatory reaction. It is well established, that depletion of MyD88 disturbs the formation of atherosclerotic plaques and reduces macrophage recruitment in the arterial wall [11–13], and thus a pro-atherosclerotic effect upon TLR9 stimulation is anticipated.

The biological effects induced by TLR9 activation seem to be dose-dependent, and published data investigating TLR9 stimulation in mice varies greatly from 10μg/kg body weight (BW) peer week to 12100μg/kg BW CpG ODN [10,14–19]. Recently, deep sequencing and micro biome data from atherosclerotic plaques have detected a much larger number and variety of bacterial and viral DNA than previously assumed [17,20–22], and TLR9 agonists may be more prevalent than estimated. We therefore analyzed the biological effects of a specific TLR9 activation at high and low CpG ODN concentrations in an acute and chronic vascular injury model in mice.

## Material and Methods

### Ethics statement

All animal experiments were performed in accordance with the Directive 2010/63/EU of the European Parliament. All animal experiments were approved by the local ethics committee of the University of Bonn (Permit Number: 84–2.04.2013.A197), supervised by the regulatory authority of the state of Nordrhein Westfalen and performed in compliance with German animal protection law.

### Animals

We used eight to twelve week old C57BL/6J wild-typ (WT) mice (Charles river) and ten week old Apolipoprotein E-deficient (ApoE^-/-^) C57BL/6 mice (Charles River) for this study. All animals (n = 99) were maintained in a 22°C room with a 12-hour light/dark cycle, and received food and drinking water *ad libitum*. To determine the required CpG ODN concentration for a proinflammatory response, WT mice were injected intravenously (i.v.) with 3nM, 10nM, or 30nM CpG ODN type B (TLR9 ligand: ODN 1826; 5’-TCCATGACGTTCCTGACGTT-3’; Invivogen, Toulouse, France) suspended in 200μl phosphate buffered saline (PBS) or vehicle (PBS). Plasma was collected two hours after incubation. To compare subcutaneous (s.c.) versus i.v. injections, WT mice were stimulated with 18nM ODN 1826 or PBS, and plasma was collected 2 to 6 hours after stimulation. For the acute injury model, 0.625nM (2.5μg/mouse), 6.25nM (25μg/mouse), 18nM (72μg/mouse), and 30nM (120μg/mouse) ODN 1826 was injected every 48 hours for a total of 4 injections. For analysis of atherosclerotic plaque development, ApoE^-/-^ mice received cholesterol-rich diet that contained 21% fat, 19.5% casein, and 1.25% cholesterol (Ssniff) for a total of seven weeks, and were concomitantly injected s.c. with either 18nM ODN 1826 suspended in 200μl PBS or vehicle every other day for six weeks. To compare the concentration dependent effect of CpG ODN, ApoE^-/-^ mice were fed a cholesterol-rich diet with 21% fat, 19.5% casein, and 1.25% cholesterol for a total of eight weeks, and were concomitantly injected s.c. with either 0.625nM or 18nM ODN 1826 suspended in 200μl PBS or vehicle every other day for the last seven weeks. All tissue and blood samples were collected and processed immediately after sacrifice.

### Carotid artery injury

Carotid artery injury was performed as previously described [23] on day three of ODN 1826 or vehicle treatment after the second CpG ODN injection. All mice (n = 60) were anesthetized with intraperitoneal injections of 150mg/kg body weight ketamine-hydrochloride (Ketanest, Riemser) and 16mg/kg body weight xylazinehydrochloride (Rompun 2%, Ceva), and all efforts were made to minimize suffering. Respiration rate, muscle relaxation, and different reflexes were used to indicate the adequacy of anesthesia. A small incision from the cranial apex of the sternum to just below the mandible was made. After careful preparation of an approximately 6 mm long segment proximal of the bifurcation, the common carotid artery was electrically denuded. A 4 mm long lesion was made by applying two serial 5 second bursts of 2 Watt using a 2 mm wide forcep. The skin was then sutured and the mice allowed to recover in individual cages before returning to their littermates. On day 8 of ODN 1826 treatment, blood was taken and 50 μl Evan’s blue solution (5%, Sigma) was injected i.v. and allowed to circulate for 2 minutes. The mice were then sacrificed and both common carotid arteries fully excised. The arteries were rinsed in 0.9% sodium chloride solution and the residual connective tissue carefully removed. Images were taken, and the total lesion area (4 mm) and remaining denuded area (stained blue) measured using AxioVision version 4.8.2 software (Zeiss). Reendothelialization is expressed by illustrating the remaining denuded area.

### Aortic ring preparations and tension recording

Vasodilation and vasoconstriction of isolated aortic ring segments were determined in organ baths filled with oxygenated modified Tyrode buffer (37°C), as previously described [23]. All visible adventitial tissue was carefully removed, and 3 mm segments of the thoracic aorta were investigated. A resting tension of 10mN was maintained throughout the experiment. Drugs were added in increasing concentrations in order to obtain cumulative concentration-response curves: KCl 20 and 40mmol/l, phenylephrine 1nmol/l to 10μmol/l, carbachol 10nmol/l to 100μmol/l (assessment of endothelium-dependent vasodilation after precontraction with phenylephrine), and nitroglycerin 1nmol/l to 10μmol/l (assessment of endothelium-independent vasodilation after precontraction with phenylephrine). The drug concentration was increased when vasoconstriction or vasorelaxation was completed. Drugs were washed out before the next substance was added.

### Measurement of reactive oxygen species (ROS)

The release of superoxide in intact aortic segments was measured by L-012 chemiluminescence, as previously described [24]. Aortas were carefully excised and placed in chilled, modified Krebs-HEPES buffer (pH 7.4; in mmol/L: NaCl 99.01, KCl 4.69, CaCl_2_ 1.87, MgSO_4_ 1.20, Na-HEPES 20.0, K_2_HPO_4_ 1.03, NaHCO_3_ 25.0, and D(+)Glucose 11.1). Connective tissue was removed and aortas were cut into 2 mm segments. Chemiluminescence of aortic segments was assessed in scintillation vials containing Krebs-HEPES buffer with 100μmol/l L-012 over 10 minutes in a scintillation counter (Lumat LB 9501, Berthold) in 1 min intervals. The vessel segments were then dried and dry weight was determined. ROS release is calculated as relative chemiluminescence per mg aortic tissue and as percent of control.

### Cytokine quantification

Concentration of Interleukin-6 and RANTES was determined in plasma of CpG ODN 1826 stimulated WT- and ApoE^-/-^-mice by ELISA. Commercially available kits for mice were used according to the manufactures protocols (Qiagen and eBioscience).

### Flow cytometry

Blood samples (50μL per mouse) were incubated with Fc-block for 5 minutes at room temperature, and then antibodies for 30 minutes at room temperature, followed by red blood cell lysis for 10 minutes.

For intracellular cytokine staining, splenocytes were counted with the Cell Counter Z2 (Beckman Coulter) and 250 000 cells were incubated with leucocytes activation cocktail (BD Bioscience) in complete RPMI (10% fetal calf serum and antibiotics) for 6 hours. After washing, cells were incubated with Fc-block for 5 minutes. Zombie Aqua (Biolegend) was used for live/dead cell differentiation for 10 minutes. Antibodies were then added and incubated another 20 minutes. Cells were fixed and permeabilized with the Intracellular Fixation & Permeabilization Buffer Set (eBioscience) according to the manufacturer’s protocol. The antibodies for intracellular cytokines were incubated for 30 minutes at room temperature in the dark.

For EMP quantification in vivo, plasma from WT mice was stained for 45 minutes with anti–CD31 APC (Becton Dickinson) and 15 minutes with anti–Annexin V (Becton Dickinson).

The following antibodies were used: anti–sca-1 FITC (Becton Dickinson), anti–vascular endothelial growth factor receptor-2 PE (VEGFR2/flk-1, Becton Dickinson). anti–CD3 PerCp-Cy5.5 (eBioscience), anti–CD4 PE-Cy7 (eBioscience), anti–CD8a Alexa Fluor 700 (BD Bioscience), anti–IFN-γ FITC (BD Bioscience), anti–CCL5/Rantes PE (Biolegend), anti–CD11b FITC (eBioscience), anti–CD45R/ B220 (BD Bioscience), anti–B220 APC (BD Bioscience), anti–CD115 PE (eBioscience), anti–NK1.1 PE-Cyanine7 (eBioscience), anti–CD289 (TLR9) FITC (eBioscience).

Analysis was performed with FACSCalibur or FACSCanto (Becton Dickinson). Data were analyzed with CellQuest (Becton Dickinson) or FlowJo (Treestar) software. Gating strategies for all of the flow cytometry panels can be found in Supporting Information S2, S5 and S7 Figs.

### Histological and immunohistochemical analysis of atherosclerotic plaques

For histological analysis of atherosclerotic plaques, hearts with ascending aortas were embedded in tissue TEC (OCT embedding medium, Miles, Elkhart, USA) snap-frozen, and stored at -80°C. Samples were sectioned on a Leica cryostat (6 μm), starting at the apex and progressing through the aortic valve area into the ascending aorta and the aortic arch, and then placed on poly-L-lysine coated slides. For the detection of atherosclerotic lesions and macrophage accumulation, aortic cryosections were fixed with 3.7% formaldehyde for 1h, rinsed with deionized water, stained with oil red O working solution (0.5%) for 30 min, and were rinsed again. Hematoxylin eosin and van Gieson staining was performed according to standard protocols. For immunohistochemical analysis of macrophages, slides were incubated with acetone for 30 min at -20°C. PBS-washed slides were preincubated with 10% normal goat serum (NGS, Sigma, St. Louis, USA) for 30 min each at room temperature (RT). The primary antibody (MOMA-2, 1:400, rat; Acris) diluted in 1% NGS was applied for 1 h at RT and then at 4°C overnight. 0,1M Tris-buffer slides were incubated with the alkaline phosphatase-conjugated secondary antibody (Sigma) for 1 h at RT. Color reaction was accomplished with FastRed (Sigma) as a chromogenic substrate. Nuclei were counterstained with Hematoxylin (blue). For immunohistochemical analysis of TLR9, slides were fixed with 4% formaldehyde for 10 minutes and subsequently incubated in Tween-20 for 5 minutes at RT. PBS-washed slides were then preincubated with 5% normal serum for 1 hour at RT. The primary antibody (TLR9, Biotin, 1:25, mice; ABIN2376552) was diluted in 1% normal serum and applied for 1 h at RT. PBS-washed slides were finally incubated with the secondary antibody (Streptavidin protein, Texas red, 1:100, ABIN459303) in normal serum for 1 h at RT. Sections were washed and mounted with DAPI (Vector Laboratories, Peterborough, UK) for light microscopic analysis. Isotype-specific antibodies were used for negative controls. Sections were washed and mounted with Aquatex mounting medium (Sigma) for light microscopic analysis. For quantification of atherosclerotic plaque formation in the aortic root, lipid-staining area and total area of serial histological sections were measured. Atherosclerotic data are expressed as lipid-staining area in percent of total surface area. The investigators who performed the histological analyses were blinded to the treatment of the respective animal group. All sections were examined under a Zeiss Axiovert 200 M microscope using AxioVision version 4.8.2 software.

### Cell culture

Human coronary artery endothelial cells (HCAEC) (Lonza, Basel, Switzerland) were cultured at 37°C and 5% CO_2_ atmospheric concentration on 6 cm dishes in endothelial cell growth medium (Promocell, Heidelberg, Germany). Experiments were performed with cells of passages 7 to 8 when grown to 70–80% confluence. For stimulation in 24-well plates, 1x10^5^ cells were incubated with 1000nM CpG ODN 2006 (Invivogen, Toulouse, France) or vehicle in cell culture medium for 24 hours.

### Real-time PCR

For analysis of gene expression in cultured HCAEC, cells were lyzed in Trizol (Invitrogen, Darmstadt, Germany) and RNA was isolated with peqGOLD RNA-Pure (peqLAB Biotechnology, Erlangen, Germany). RNA concentration and quality was verified with a spectrophotometer. 1 μg of the isolated total RNA was reversely transcribed using Omniscript RT Kit (Qiagen) according to the manufactures protocol. The single-stranded cDNA was amplified by real-time quantitative reverse transcription-polymerase chain reaction (RT-PCR) with the TaqMan system (ABI-7500 fast PCR System) using TaqMan gene expression assay probes specific for MyD88 (Hs01573837_g1, Applied Biosystem, Warrington, United Kingdom), IRAK2 (Hs00176394_m1, Applied Biosystem) and IRAK4 (Hs00928779_m1, Applied Biosystem) or individual primers and SYBR-Green detection dye. Primers used: ICAM-1 forward 5’-CGCAAGGTGACCGTGAATGT-3’, reverse 5’-CGTGGCTTGTGTGTTCGGTT-3’; VCAM-1 forward 5’-AGTCAGGAATTTCTGGAGGATGC-3’, reverse 5’-GCAGCTTTGTGGATGGATTCAC-3’; 18s forward 5’-GTAACCCGTTGAACCCCATT-3’; reverse 5’-CCATCCAATCGGTAGTAGCG-3’. For quantification, mRNA expression was normalized to endogenous 18s rRNA.

### Statistical analysis

Data are presented as mean±standard error of mean (SEM). For statistical analysis, 2-tailed, unpaired Student's t-test and ANOVA for multiple comparisons were employed where applicable as indicated. P<0.05 indicates statistical significance.

## Results

The aim of our study was to explore the potential role of toll-like receptor 9 in acute and chronic vascular injury mouse models.

### Acute vascular injury

**Fig 1:**
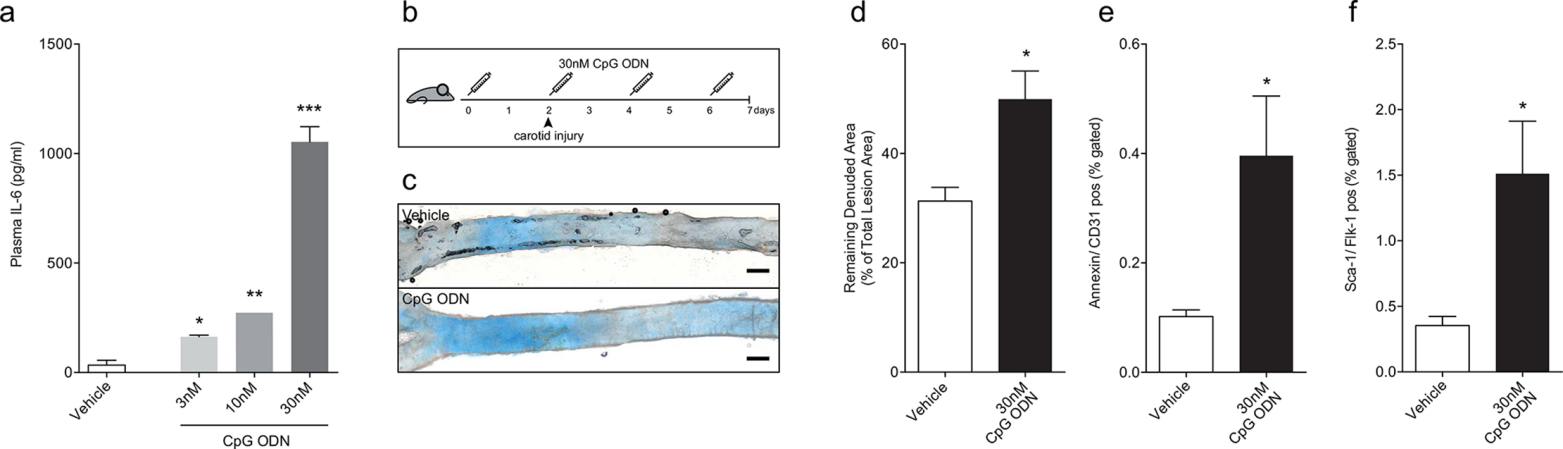
CpG ODN stimulation in acute vascular injury. WT mice were injected with PBS (n = 4) or 3-30nM CpG ODN (n = 2) i.v., respectively. Activation of TLR9 with 30nM CpG ODN led to the highest plasma IL-6 concentrations in mice compared to vehicle controls (a). For the acute injury, WT mice (n = 12–13) were subjected to an electric denudation of the left carotid artery and received repetitive injections of 30nM CpG ODN or vehicle over 7 days (b). Reendothelialization was greatly impaired in TLR9-stimulated mice compared to vehicle controls (c, representative Evan’s blue stained photomicrograph; and d, quantitative). The number of circulating EMPs (e, n = 5) and the number of circulating sca-1/flk-1 positive cells (f, n = 5) were significantly increased in ODN treated mice. Gating strategy for FACS analysis is shown in S3 Fig. Data are presented as mean±SEM. Statistical analysis was performed using 2-tailed, unpaired students t-test. Scale bar 200μm in c. *p<0.05, **p<0.001, ***p<0.0001.

To determine the optimal concentration of CpG ODN 1826 to sufficiently induce a proinflammatory TLR9 mediated systemic response, we first injected C57Bl/6 mice (WT) with 3 to 30nM CpG ODN intravenously (i.v.) and measured plasma Interleukin-6 (IL-6) concentrations. Fig 1A illustrates a dose-dependent IL-6 induction and 30nM CpG ODN led to the highest IL-6 formation. For all CpG ODN dosages there were no evident signs of side effects; body weight, food, and water intake remained unchanged.

Next, we explored the potential effects of TLR9 activation in an acute vascular injury model. For this, WT mice were treated with 30nM CpG ODN every other day, subjected to an electric denudation of the left common carotid artery on day two, and reendothelialization was quantified after 5 days (Fig 1B). TLR9 activation induced a systemic inflammatory response by significant induction of IL-6 and RANTES (S1 Fig). As a result, vascular reendothelialization was impaired by 54% in TLR9 stimulated mice compared to vehicle controls (Fig 1C and 1D). The number of circulating endothelial microparticles (EMP), a marker of endothelial damage in mice (Fig 1E, S2 Fig), and the number of circulating sca-1/flk-1 positive cells (Fig 1F, S2 Fig) were significantly increased in these TLR9 stimulated mice. Interestingly, TLR9 activation did not influence endothelium-dependent vasodilation of intact aortic segments, suggesting that only regenerative and not vasoactive functions of the endothelium are affected by TLR9 induced inflammation (S3 Fig).

### Concentration dependent vascular injury

Because others had proposed a vasoprotective role for CpG ODN at much lower concentrations and alternate application forms, we sought to investigate whether our observed findings are merely dose-dependent or due to the i.v. injection. First, to compare s.c. and i.v. delivery WT mice received either 18nM CpG ODN or vehicle by s.c. or i.v. injection. IL6 plasma response was identical in both s.c. and i.v. treated mice (S4 Fig).

**Fig 2:**
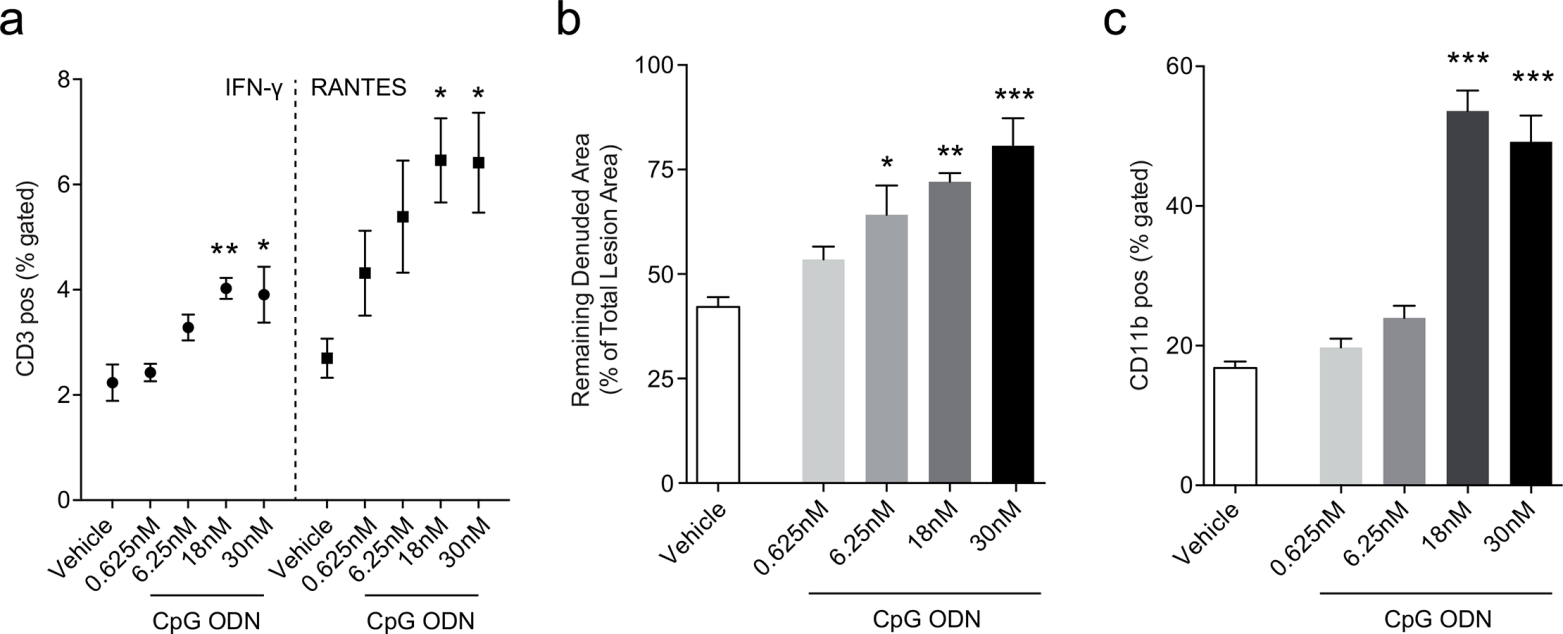
Proinflammatory response by concentration dependent TLR9 activation in acute vascular injury. WT mice were subjected to an electric denudation of the left carotid artery as described in Fig 1 and received repetitive s.c. injections of 0.625nM (n = 7), 6.25nM (n = 7), 18nM (n = 6), 30nM CpG ODN (n = 7) or vehicle (n = 7) over 7 days. Splenic CD3 positive lymphocytes from 18nM and 30nM CpG ODN treated mice expressed more IFN-γ and RANTES compared to vehicle controls (a). TLR9 activation by 6.25nM, 18nM and 30nM CpG ODN significantly impaired reendothelialization in WT mice compared to vehicle controls (b). Further, stimulation with 18nM and 30nM CpG ODN elevated levels of circulating CD11b positive cells (c). Data are presented as mean±SEM. Statistical analysis was performed using ANOVA (Holm-Sidak's multiple comparisons test). *p<0.05, **p<0.001, p***<0.0001.

Next, we tested increasing CpG ODN doses ranging from 0.625nM to 30nM in our acute vascular injury mouse model. In a flow cytometry based approach we found that viable B- and T- lymphocytes express both IFN-γ and RANTES in response to leukocyte activation cocktail (S5 Fig). T-lymphocytes (CD3^+^) of WT mice stimulated with 18nM and 30nM CpG ODN expressed significantly more IFN-γ and RANTES compared to cells from vehicle treated mice (Fig 2A), whereas only RANTES was increased in B-lymphocytes from CpG ODN treated mice (S6 Fig). This indicates that high doses of CpG ODN results in a proinflammatory conditioning of lymphocytes. Importantly, histological analysis of the carotid artery revealed that TLR9 activation with 6.25nM, 18nM and 30nM CpG ODN significantly impaired reendothelialization compared to vehicle controls (Fig 2B). None of the doses tested resulted in improved endothelial rejuvenation.

Because CpG ODN induced the mobilization of bone marrow derived sca-1/flk-1 positive cells in our previous experiment, we investigated whether the number of circulating leukocytes is also affected (S7 Fig). Differential blood count analysis revealed a reduction of B-lymphocytes without consistently affecting T-cells or natural killer cells (S8 Fig). Interestingly, CpG ODN treatment greatly induced the number of proatherosclerotic CD11b positive cells (Fig 2C). As has been previously described [25], CpG ODN stimulation led to splenomegaly in a concentration dependent matter (S9 Fig).

### Chronic vascular injury

**Fig 3:**
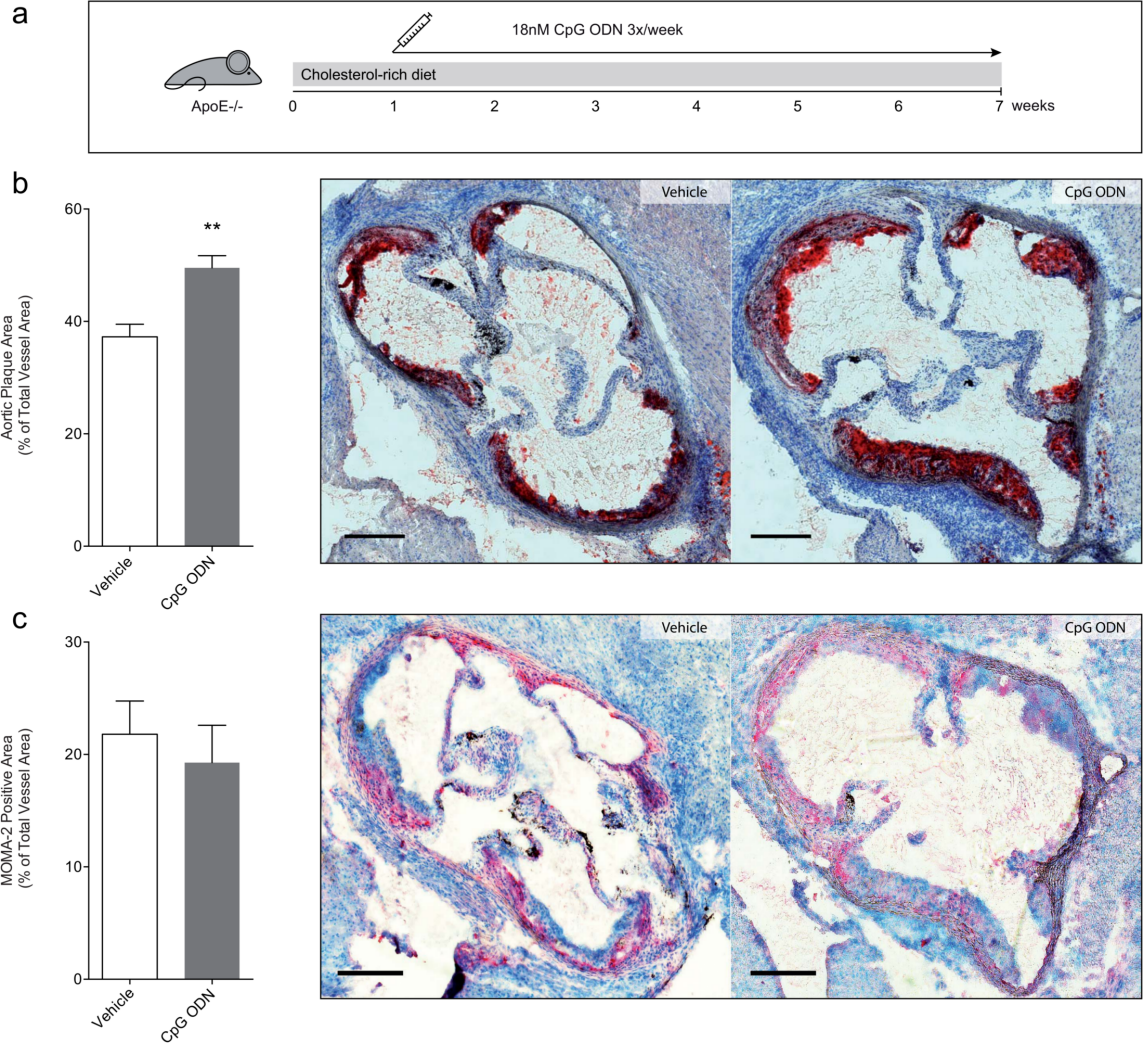
TLR9 stimulation augments atherosclerosis. ApoE^-/-^ mice were fed a cholesterol rich diet for 7 weeks and treated 3 times a week with 18nM CpG ODN s.c. for 6 weeks (a). Chronic stimulation of TLR9 in ApoE^-/-^ mice led to a significant increase in aortic atherosclerotic plaque size compared to vehicle treated mice (b, quantitative and representative Oil-red O image; n = 9). MOMA-2 stained atherosclerotic plaque were similar compared to vehicle treated mice (c, quantification and representative image). Data are presented as mean±SEM. Statistical analysis was performed using 2-tailed, unpaired students t-test. Scale bar 200μm in b and c. **p<0.001.

We next investigated proinflammatory TLR9 stimulation in a chronic vascular injury model. For this, ApoE^-/-^ mice were fed a cholesterol-rich diet and, to avoid mouse tail injury, concomitantly injected s.c. with either 18nM CpG ODN or vehicle 3 times per week for six weeks (Fig 3A). TLR9 stimulation drastically augmented the formation of atherosclerotic plaques and increased the lipid stained area in aortic roots by 39% compared to vehicle treated mice (Fig 3B). Although, plaque cellularity and density are slightly reduced in CpG ODN treated mice (S10 Fig), we did not detect a relevant change in connective tissue (S10 Fig) or macrophage infiltration (Fig 3C).

As in the acute injury model, increased EMP levels were detected by chronic CpG ODN treatment (S11A Fig), and the number of circulating sca-1/flk-1-positive cells was elevated (S11B Fig) suggesting increased endothelial cell turnover. Because ROS formation is induced by TLR9 and can contribute to atherogenesis by activation of endothelial cells, we further measured ROS production in aortic segments of these mice. Mice treated with CpG ODN displayed increased vascular ROS formation compared to control animals (S11C Fig). Finally, because hypercholesterolemia is the driving force of plaque development in this model we measured cholesterol concentrations in our mice. Both CpG ODN and vehicle treated mice had similarly elevated cholesterol levels indicating that cholesterol lowering is not involved in this TLR9 mediated effect (S11D Fig). Of note, both cholestanol, a marker of cholesterol absorption, and Lathosterol, an indicator for cholesterol synthesis, were significantly reduced by CpG ODN, while both plants sterols Campesterol and Sitosterol were not affected (S11E Fig).

**Fig 4:**
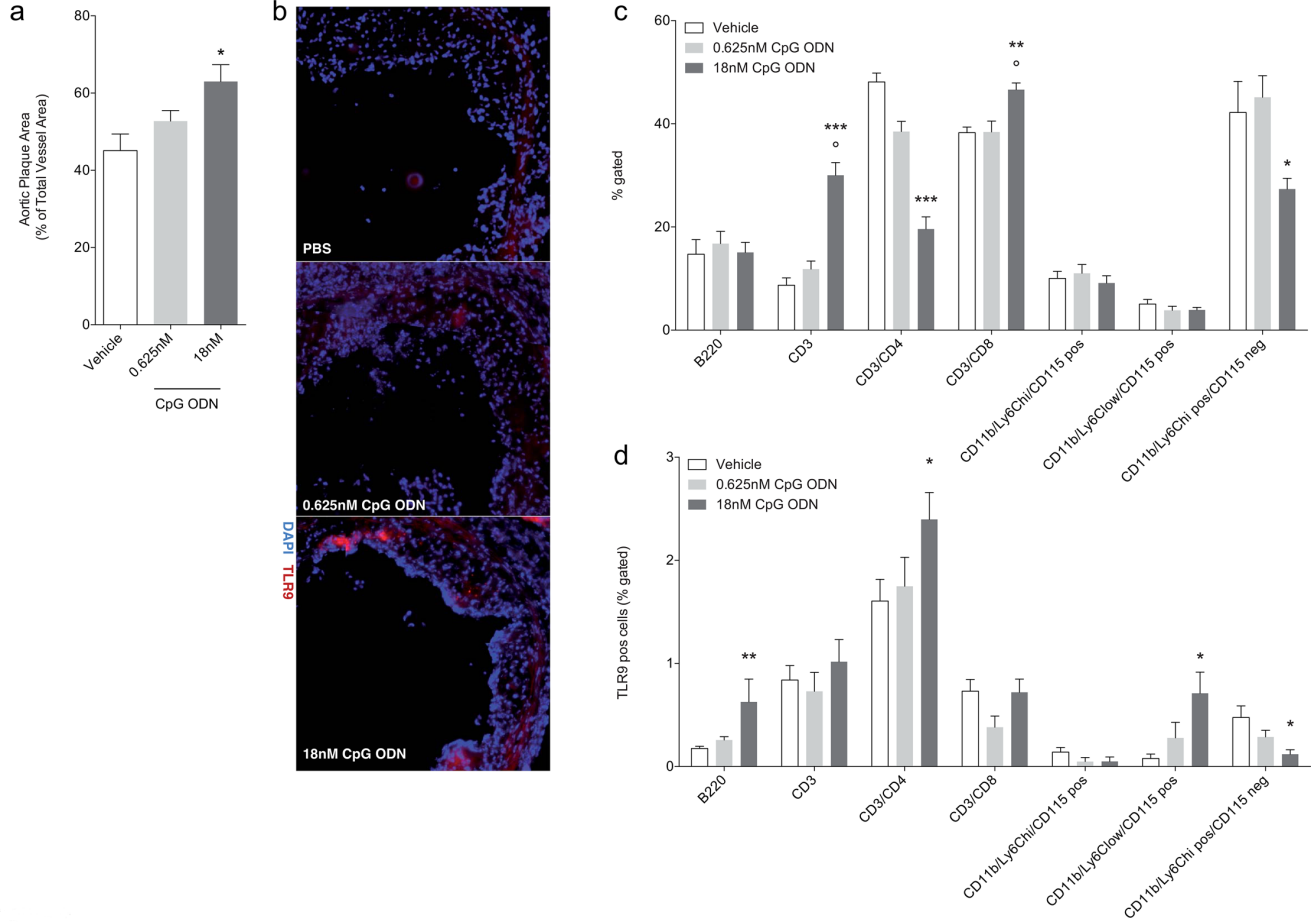
Concentration dependent plaque development by TLR9 stimulation. ApoE^-/-^ mice were fed a cholesterol rich diet for 8 weeks and treated 3 times a week s.c. with 0.625nM (n = 7) or 18nM CpG ODN (n = 7), or vehicle (n = 8) in the last 7 weeks. Chronic TLR9 stimulation with high concentrations of CpG ODN in ApoE^-/-^ mice led to a significant increase in aortic atherosclerotic plaque size compared to low dose CpG ODN and vehicle treated mice (a). Immunohistochemical staining demonstrates increased TLR9 expression in aortic plaques of 18nM CpG ODN treated mice (b, representative image). Differential blood analysis shows increased CD3 and CD3/CD8 positive cells, while CD3/CD4 and neutrophil granulocytes (CD11b^+^/Ly6C^+^/CD115^-^ cells) are reduced (c). TLR9 expression is induced in B-cells, T-helper cells, and non-classical monocytes (CD11b^+^/Ly6C^+^ low/CD115^+^) are by CpG ODN, while it is decreased in neutrophil granulocytes (d). Data are presented as mean±SEM. Statistical analysis was performed using Kruskal-Wallis test (Dunn’s multiple comparison test). *p<0.05, **p<0.001, ***p<0.0001, ˚p<0.05 18nM to 0.625nM CpG ODN.

To evaluate if there is indeed a dose dependent effect of TLR9 activation in our chronic vascular injury model, ApoE^-/-^ mice were fed a cholesterol-rich diet for 8 weeks and concomitantly injected s.c. with either 0.625nM (10μg/week as previously described by Koulis et al.) or 18nM CpG ODN or vehicle 3 times per week for the last seven weeks. As expected, TLR9 stimulation with 18nM CpG ODN significantly increased the formation of atherosclerotic plaques compared to vehicle treated mice (Fig 4A). Treatment with 0.625nM CpG ODN however did not significantly affect the formation of atherosclerotic plaques in our trial. TLR9-expression in atherosclerotic plaque was analyzed by immunohistochemistry and revealed an increase only in 18nM CpG ODN treated mice (Fig 4B). We further investigated whether differential blood was affected. High dose CpG ODN stimulation elevated T-lymphocytes and CD3/CD8 positive cells, while repressing T-helper cells and neutrophil granulocytes (Fig 4C). B-cells, classical and non-classical monocytes were not affected. Interestingly, TLR9-expression was induced in B-cells, T-helper cells and non-classical monocytes upon TLR9 stimulation with high dose CpG ODN while the amount of TLR9 was repressed in neutrophil granulocytes (Fig 4D). Of note, low dose CpG ODN did not significantly affect any of the measured parameters.

Finally, to analyze the mechanistic pathway of TLR9-activation, we stimulated human coronary artery endothelial cells (HCAEC) with 1000nM CpG ODN 2006. TLR9-activation significantly induced IRAK2 mRNA expression levels, but did not affect MyD88, IRAK4, ICAM-1 or VCAM-1 mRNA expression levels (S12 Fig). The complex signaling cascade of TLR9 is shown in S12F Fig. All raw data of the figures can be found in S13 Fig.

## Discussion

In the present study we report that a proinflammatory stimulation of TLR9 impairs reendothelialization following acute vascular injury and increases plaque development in ApoE^-/-^ mice.

TLR9 is activated by CpG motifs in ODN and there are 3 distinct types of CpG ODN (A-, B- and C-class) that induce specific responses by the innate and adaptive immune system [26,27]. In our experiments, we used B-class CpG ODN 1826, a potent Th1 adjuvant with predominant B-cell-activating effects. It also effects plasmacytoid dendritic cells and anti-tumor activity, is known to activate macrophages, and promotes cytokine secretion [28,29] via stimulation of the gene transcription factors NF-κB and interferon regulatory transcription factor (IRF) 7 [30]. While B-class CpG ODN stimulate pDC to produce moderate amounts of type I interferons, A-class CpG ODN stimulate very high levels of IFN-alpha and IFN-beta. Through this, A-class CpG ODN are weak in mediating other TLR9 dependent effects such as B-cell stimulation. C-class CpG ODN on the other hand have intermediate immune effects compared to B- and A-class, with efficient effects on co-stimulatory molecules, inducing B-cell IL-6 or IL-10 production, and stimulating strong B-cell proliferation [27,31]. Upon ligand binding and TLR9 activation, the MyD88 death domain assembles in a helical signaling oligomer consisting of MyD88, IL-1 receptor-associated kinase (IRAK) 4 and IRAK2 death domains. This signaling complex, the so-called myddosome, assembles in a hierarchical manner so that IRAKs can associate with tumor necrosis factor receptor-associated factor 6 (TRAF6), which, via a complex signaling cascade, ultimately enables NF-κB to translocate into the nucleus where it regulates gene transcription [8,32]. TRAF6 can also associate with the IKK-related kinases, tank binding kinase 1 and IKKε [33]. This in turn facilitates the nuclear translocation of IRF-3 and induces the production of type 1 interferons. MyD88 is therefore the central protein for TLR9 signal transduction [34].

MyD88 deficient mice display reduced atherosclerotic plaque formation and impaired macrophage recruitment to the artery wall [12]. As expected, stimulation of TLRs with a MyD88-dependent signal transduction pathway, such as TLR2 and 4, enhances atherogenesis, and TLR-knock out mice show an atheroprotective phenotype [8]. This is in conjunction with our findings.

Koulis et al. recently published interesting data suggesting an anti-atherosclerotic role of TLR9. ApoE^-/-^/TLR9^-/-^ mice had a significant 33% increase in atherosclerotic plaque size compared with ApoE^-/-^ mice. Immunostimulatory concentration of ODN 1668 (10μg/mouse per week) further attenuated atherosclerotic lesion development. Contradictory data with pro- and anti-inflammatory effects in response to TLR9 stimulation have even been described before. On the one hand, TLR9 stimulation protects cardiomyocytes from stress by inhibition of SERCA2 (sarco/endoplasmic reticulum calcium ATPase) [35], attenuates cardiac dysfunction in sepsis [36], mediates cardioprotection by reducing infarct size in a postconditioning manner [14], and plays a protective role in Th1 modulation and in deep vein thrombogenesis [17]. In autoimmune diseases such as systemic lupus erythematosus, deletion of TLR9 led to exacerbation of the disease [37], CpG ODN decreased I/R-induced infarct size and improved cardiac function following myocardial I/R [38], and finally Koulis et al. showed a protective role for TLR9 in atherosclerosis [10]. On the other hand, several reports indicate a proatherosclerotic role of TLR9. High-mobility group box 1 (HMGB1) is a proinflammatory mediator and protein located in the cell nucleus. Hirata et al. demonstrated the involvement of High-mobility group box 1 and TLR9 in vascular remodeling in a neointima injury model with induced cytokine production in response to CpG ODN [39]. TLR9 is involved in hemorrhagic shock-induced proinflammatory response [40] and treatment with TLR9 antagonist in apoE*3-Leiden mice lead to reduced macrophage infiltration and reduced neointima formation [41]. Boehm and colleges could further show, that systemic TLR9 stimulation is able to depress cardiac function [15]. These reports highlight the complex and still poorly understood functions of TLR9 in vascular injury and inflammation.

The discrepancy between our findings and the data published by Koulis et al. could be explained by the differences in CpG ODN sequence and dosing regime. As in our experiments, Koulis et al. also used a B-class CpG ODN but with a different base sequence (ODN 1668). Although it is unlikely that this could be responsible for our opposing findings, especially since no significant differences in the biological effects between ODN 1826 and 1668 have been reported [42], we cannot fully exclude the possibility. To induce chronic inflammation, we injected our mice with cumulatively 10 or 216μg/week (in three individual injections) while Koulis performed one injection with 10μg/week. As published by Boehm et al., CpG ODN dosage appears to play an important role in TLR9 activation. They and others could demonstrate that low concentrations (0.25nmol/g) serve as a mild stimulus for cardiac preconditioning, and higher concentrations (0.5 to 1nmol/g) induced a sepsis-like inflammatory response [15,43–45]. In our acute vascular injury experiments we tested a wide rage of doses. The lowest CpG ODN stimulation (0.625nM), comparable to the dose used by Koulis et al. [29], showed no trend towards enhanced reendothelialization. Indeed, CpG ODN consistently impaired reendothelialization in a dose dependent manner, indicating a TLR9 mediated detrimental vascular effect. Even at high CpG ODN 1826 concentrations we did not observe serious adverse effects. This is consistent with findings by Davis et al. who investigated the toxicity of CpG ODN and found that injections with concentrations of 10–500μg per mouse, four times higher than what we used in our experiments, did not produce toxic effects [26]. The time intervals between injections might also be relevant. In all our experiments, including the final direct comparison of low and high dose CpG ODN on atherosclerotic plaque development, we performed repetitive injection 3 times per week while Koulis et al. injected mice once a week. Atherogenesis however requires a chronic vascular inflammation that can only be attained by “constant” stimulation. Because the number and variety of bacterial and viral DNA found in atherosclerotic lesions by deep sequencing studies greatly surpasses what has been previously assumed using microbiological investigations [22,46] our dosing regime may be more representative of actual physiological conditions during atherogenesis.

Sorrentino [47] and Lee [48] have demonstrated that TLR9 activation of macrophages by CpG ODN stimulation induces foam cell formation via involvement of the liver X receptor. Although, we do not find a direct increase in the number of plaque infiltrating monocytes in our chronic injury model, only an increase in TLR9 stained non-classical monocytes, these results are consistent with the general proatherogenic theory of TLR9 mediated effects. Furthermore, we did find an increase lipid accumulation in CpG ODN treated mice as assessed by Oil-Red-O staining that could be the result of increased foam cells formation.

The inflammatory response in mice, induced by TLR9 activation leads to elevated concentrations of CXCL2, TNF-α, CCL2, IFN-γ, IL-6, IL-12, IL-18 and the anti-inflammatory cytokine IL-10 [18,49]. Behrens et al. have shown an IFN-γ dependent macrophage activation syndrome-like disease by repetitive i.p CpG ODN injections (50μg), without requirement of exogenous antigen or adaptive immunity [49]. Most recently, Behrens et al. could further demonstrate, that TLR9 and IFN-γ are acting independently in reducing B-cells by *in vivo* CpG ODN stimulation [50]. CpG ODN induced B-cell depletion might therefore be responsible for the systemic inflammatory reaction promoting atherosclerosis [37]. Consistently, we measured reduced B-cells in the acute injury with elevated plasma levels of IL-6 and RANTES as well as increased expression of IFN-γ and RANTES by splenic lymphocytes.

It is well established that the number of circulating proatherogenic monocytes/ macrophages is increased in inflammation. The elevated levels of CD11b positive cells in our acute vascular injury model confirm this. Interestingly, blockade of TLR9 leads to reduced macrophage activation and foam cell formation during arterial restenosis [41].

In conclusion, we find that systemic stimulation of TLR9 by CpG ODN 1826 leads to impaired reendothelialization upon acute vascular injury and is associated with the production of proinflammatory cytokines, and increased formation of circulating EMPs. Importantly, ApoE^-/-^ mice chronically treated with high concentration of a TLR9 agonist displayed increased atherosclerotic plaque development. Our data highlight the importance of fully understanding the pathomechanisms involved in innate immune activation during atherogenesis. To address the contradictory results of TLR9 agonism, particularly in atherogenesis, further experiments are warranted assessing the effects of specific TLR9 agonists at different concentrations.

## Supporting Information

S1 Fig Cytokine concentration in acute vascular injury. WT mice were treated as described in the acute injury model. Plasma cytokine concentration was measured by ELISA. 30nM CpG ODN stimulation significantly elevated plasma levels of IL-6 (n = 3) and RANTES (n = 6–7) compared to vehicle control. Data are presented as mean±SEM. Statistical analysis was performed using 2-tailed, unpaired students t-test. *p<0.05.
(EPS)

S2 Fig Flow cytometric gating strategy for EPCs and EMPs. Following red blood cell lysis, viable lymphocyte population was gated and assessed for the expression of sca-1 and flk-1 positive cells (a). To investigate endothelial microparticles (EMPs), cell free plasma of WT mice was assessed for particles expressing Annexin V and CD31 (b).
(EPS)

S3 Fig Endothelial vasoactive function in acute vascular injury. Vasodilation and vasoconstriction of isolated aortic ring segments was determined in organ baths to evaluate endothelial function after acute vascular injury (n = 9). After precontraction with phenylephrine (a), endothelium-dependent vasodilation by carbachol (b) and endothelium-independent vasodilation by nitroglycerin (c) was measured. No significantly differences between the groups was observed. Data are presented as mean±SEM. Statistical analysis was performed using 2-tailed, unpaired students t-test.
(EPS)

S4 Fig Application method of CpG ODN on inflammatory response. To determine the CpG ODN effect induced by distinct application forms, WT mice were stimulated s.c. (n = 3) or i.v. (n = 3) with 18nM CpG ODN or vehicle, and plasma was collected 2, 4 and 6 hours (each n = 3) after stimulation. IL-6 plasma release upon CpG ODN injection was similar in both s.c. and i.v. groups (p>0.05). Data are presented as mean±SEM. Statistical analysis was performed using Kruskal-Wallis test (Dunn’s multiple comparison test).
(EPS)

S5 Fig Gating strategy for splenic cytokine expression. We gated viable lymphocytes for B220 positive and CD3 positive cells and assessed the expression of IFN-γ and RANTES.
(EPS)

S6 Fig Splenic B-cell cytokine expression. Acute injury in WT mice did not affect IFN-γ expression on B220 positive cells, while RANTES was significantly induced in B220 positive cells by 0.625nM-30nM CpG ODN stimulation. Data are presented as mean±SEM. Statistical analysis was performed using Kruskal-Wallis test (Dunn’s multiple comparison test). *p<0.05, **p<0.01.
(EPS)

S7 Fig Gating strategy of differential blood count. Viable cells were stained with anti-mouse B220 for B-cells, anti-mouse CD4 for T-cells, anti-mouse NK1.1 for natural killer cells, anti-mouse CD11b for macrophages. These CD11b^+^ cells were again stained for CD115^+^ and Ly6C.
(EPS)

S8 Fig Differential blood count. Blood was drawn from WT mice after acute vascular injury (n = 6–7). Treatment with 18nM and 30nM CpG ODN significantly reduced amount of B220 positive cells compared to vehicle control (a). CD4 positive cells were not affected by CpG ODN treatment (b). Natural killer cells were reduced after treatment with 6.25nM CpG ODN (c) and classical monocytes (CD11b^+^; Ly6C^+^ high; CD115^+^) were induced by 6.25nM CpG ODN (d). Non-classical monocytes (CD11b^+^; Ly6C^+^ low; CD115^+^ cells) were not affected by CpG ODN. Neutrophil granulocytes (CD115^-^) were reduced by 30nM CpG ODN (f). Data are presented as mean±SEM. Statistical analysis was performed using Kruskal-Wallis test (Dunn’s multiple comparison test). *p<0.05, ***p<0.0001.
(EPS)

S9 Fig Spleen weight of WT mice in acute injury. Spleen weight of WT mice (n = 6–7) were significantly increased by CpG ODN in a concentration depending matter. Data are presented as mean±SEM. Statistical analysis was performed using ANOVA (Dunn’s multiple comparison test). ***p<0.0001.
(EPS)

S10 Fig Van Gieson Elastica and H&E staining of atherosclerotic plaques. Histological cross sections of the mouse aortic sinus after stimulation with 18nM CpG ODN from the chronic injury model. Representative Images in the van Gieson elastica staining and Haematoxylin and Eosin staining, original magnification 10x.
(EPS)

S11 Fig Inflammation in chronic vascular injury. ApoE^-/-^ mice were fed a cholesterol rich diet for 7 weeks and stimulated with 18nM CpG ODN or vehicle over 6 weeks (n = 9). This TLR9-activation increased (a) the number of circulating endothelial microparticles and (b) sca-1/flk-1 positive cells. As a sign of vascular oxidative stress, production of reactive oxygen species (ROS) in thoracic aorta (n = 7–8) was also significantly increased in CpG ODN treated mice (c). Cholesterol levels were similar in CpG ODN and vehicle treated mice (d, n = 4–5). Cholestanol and Lathosterol levels in plasma were reduced by CpG ODN stimulation. Campesterol and Sitosterol levels were not affected in our chronic vascular injury (e). Data are presented as mean±SEM. Statistical analysis was performed using 2-tailed, unpaired students t-test. *p<0.05.
(EPS)

S12 Fig TLR9 signaling cascade. To analyze the mechanistic pathway of TLR9-activation, we stimulated human coronary artery endothelial cells with 1μM CpG ODN 2006 (n = 6). TLR9-activation significantly induced IRAK2 mRNA expression levels (b), but did not affect MyD88 (a), IRAK4 (c), ICAM-1 (d), or VCAM-1 (e) mRNA expression levels. (f) shows the TLR9 signaling pathway. Data are presented as mean±SEM. Statistical analysis was performed using 2-tailed, unpaired students t-test. *p<0.05.
(EPS)

S13 Fig Raw data of all figures. (XLSX)
